# Surgical approaches for stage I and II thymoma-associated myasthenia gravis: feasibility of complete video-assisted thoracoscopic surgery (VATS) thymectomy in comparison with trans-sternal resection

**DOI:** 10.7555/JBR.27.20120060

**Published:** 2012-11-30

**Authors:** Zhicheng He, Quan Zhu, Wei Wen, Liang Chen, Hai Xu, Hai Li

**Affiliations:** a Department of Thoracic Surgery;; b Department of Radiology;; c Department of Pathology, the First Affiliated Hospital, Nanjing Medical University, Nanjing, Jiangsu 210029, China.

**Keywords:** video-assisted thoracoscopic surgery (VATS), thymoma, thymectomy, myasthenia gravis, adjuvant pneuomomediastinum

## Abstract

Complete resection could be achieved in virtually all myasthenic patients with Masaoka stage I and II thymoma using the trans-sternal technique. Whether this is appropriate for minimally invasive approach is not yet clear. We evaluated the feasibility of complete video-assisted thoracoscopic surgery (VATS) thymectomy for the treatment of Masaoka stage I and II thymoma-associated myasthenia gravis, compared to conventional trans-sternal thymectomy. We summarized 33 patients with Masaoka stage I and II thymoma-associated myasthenia gravis between April 2006 and September 2011. Of these, 15 patients underwent right-sided complete VATS (the VATS group) by using adjuvant pneuomomediastinum, comparing with 18 patients using the trans-sternal approach (the T3b group). No intraoperative death was found and no VATS case required conversion to median sternotomy. Significant differences between the two groups regarding duration of surgery and volume of intraoperative blood loss (*P* = 0.001 and *P* < 0.001, respectively) were observed. Postoperative morbidities were 26.7% and 33.3% for the VATS and T3b groups, respectively. All 33 patients were followed up for 12 to 61 months in the study. The cumulative probabilities of reaching complete stable remission and effective rate were 26.7% (4/15) and 93.3% (14/15) in the VATS group, which had a significantly higher complete stable remission and effective rate than those in the T3b group (*P* = 0.026 and *P* = 0.000, respectively). We conclude that VATS thymectomy utilizing adjuvant pneuomomediastinum for the treatment of stage I and II thymoma-associated myasthenia gravis is technically feasible but deserves further investigation in a large series with long-term follow-up.

## INTRODUCTION

Myasthenia gravis (MG) is a neuromuscular junction disease characterized by muscular weakness and fatigability. Epidemiological data show that 10%-20% of myasthenic patients have a thymoma. On the other hand, 20%-25% patients with a thymoma have MG[1]. Virtually all myasthenic patients with thymoma can be treated using the trans-sternal approach. Whether this is true for minimally invasive approach is not yet clear.

A minimally invasive approach does not necessarily mean minimal exposure, but it provides ideal exposure and visualization of all the anterior mediastinal tissue because of the magnification and lighting associated with the video technique. It is universally accepted that the less invasive thoracoscopic approach is associated with less pain, faster recovery, superior cosmesis, and greater patient acceptance[2]. However, the role of video-assisted thoracoscopic surgery (VATS) thymectomy on the thymoma-associated myasthenia gravis (T-MG) is still under study. Many surgeons remain skeptical of this new option and do not regard it as equivalent to the trans-sternal approach which has been considered as the preferred standard treatment for MG for a long time.

In this study, we conducted a retrospective review to clarify the efficacy of a right-sided VATS thymectomy utilizing adjuvant pneuomomediastinum as an alternative treatment for Masaoka stage I and II T-MG, and compared the outcomes with conventional trans-sternal thymectomy.

## SUBJECTS AND METHODS

### Patients

We retrospectively reviewed our experiences in 64 consecutive complete thymectomies for the treatment of MG in the First Affiliated Hospital of Nanjing Medical University from April 2006 to September 2011. For the homogeneity of the cohort, only 33 cases meeting the following three inclusion criteria were finally selected for the study: 1) patients were diagnosed with MG, 2) the presence of anterior mass (indicating thymoma) was confirmed on preoperative CT scan, and 3) the thymoma was histopathologically confirmed as Masaoka I and II stage. The diagnosis of MG was mainly based on the clinical manifestations and supported by electromyographic studies or acetylcholine receptor antibody studies. CT of the thorax was routinely performed in all patients prior to surgery. No specific preoperative preparation or optimization regimen including intravenous immune globulin therapy and plasmapheresis were performed. All patients were optimally stabilized before surgery and no patient was operated on during the crisis.

The severity of MG was retrospectively evaluated at the time of diagnosis according to the MG Foundation of America (MGFA) clinical classification[3]. Modified Masaoka staging was used for thymoma clinical staging: stage 1 indicated no invasion and an intact capsule; stage 2 indicated macro- or microscopic capsular invasion into perithymic fat[4]. The Japanese Association for Research on the thymus (JART) has made a modification to the Masaoka staging system in which invasion to the mediastinal pleura (stage 2) is now determined by pathology instead of surgical observation[5]. All specimens were reviewed by pathologists and thymomas were histologically classified based on the new World Health Organization (WHO) classification[6].

Informed consent was obtained from all patients who were given information on surgical options of VATS and trans-sternal approaches and the potential complications. Patients were grouped according to their preference of treatment option. The study was approved by the institutional review board and ethics committee at the authors’ affiliated institution. Both hospital records and telephone interviews were used in data collection and subsequent analysis.

The neurological outcomes were classified according to the MGFA postoperative status and simplified as: complete stable remission (CSR) and effective rate (ER). CSR was defined as the absence of symptoms or signs of MG for at least 1 year and receiving no therapy for MG during the period. There was no weakness of any muscle on careful examination by a specialist skilled in the evaluation of neuromuscular disease. Isolated weakness of eyelid closure was accepted. ER included CSR, pharmacologic remission, minimal manifestations and improved status[3].

### Surgical technique of VATS thymectomy

**Fig. 1:**
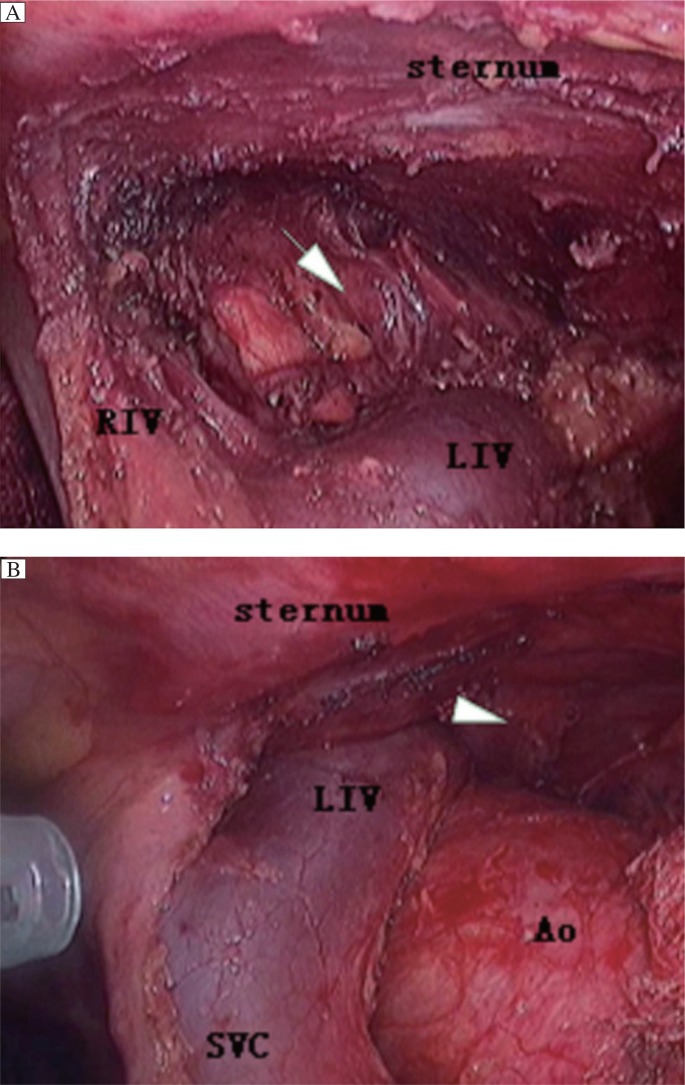
The comparative graphs of the left superior pole of the thymus without (A) and with (B) carbon dioxide insufflations during the same VATS procedure. The zone marked by arrow indicates the site of the left superior pole of thymus with carbon dioxide insufflations, which can be easily visualized and reached while the same site marked by arrowhead cannot be visualized without carbon dioxide insufflations. LIV: the left innominate vein; RIV: the right innominate vein; SVC: the superior vena cava; Ao: the ascending aorta.

**Fig. 2:**
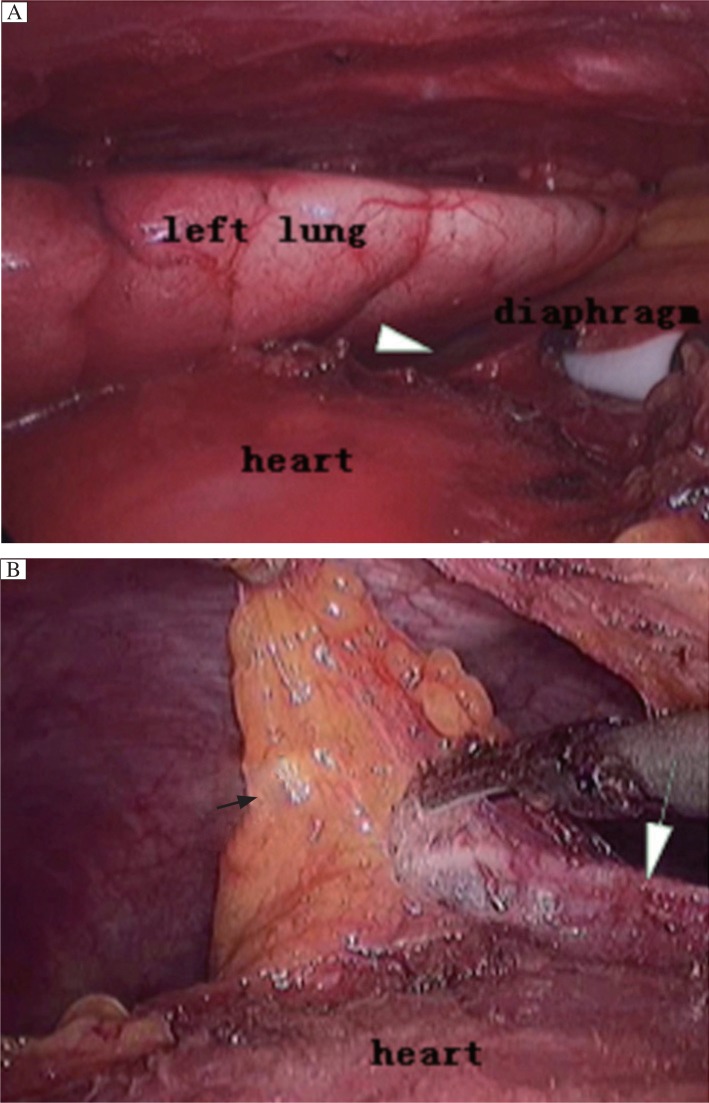
The comparative graphs of the left cardiophrenic portion without (A) and with (B) carbon dioxide insufflations during the same VATS procedure. The zone marked by white arrowhead indicates the site of the left cardiophrenic portion without carbon dioxide insufflations, which cannot be easily visualized. With carbon dioxide insufflations, the left cardiophrenic perithymic fatty tissue marked by white arrow can be easily dissected with the left phrenic nerve marked by black arrow kept intact during the VATS procedure.

For the VATS group, the patients were anaesthetized by using a double-lumen tube for split-lung ventilation and were placed up 30° in the left semi-supine position with a roll under the right shoulder. The first sealed-trocar, which allowed the introduction of 30°-angled telescope, was inserted at the 5^th^ or 6^th^ intercostal space in the mid-axillary line according to the site of thymomas. Once the thymoma was identified, two other sealed-trocars as instrumental ports were positioned in such a way as to provide the best convergence on the thymoma. They were more frequently inserted at the 3^rd^ intercostal space in the anterior-axillary line and at the 5^th^ intercostal space in the mid-clavicle line (along the inframammary fold for female patients), respectively. Persistent carbon dioxide insufflation at the pressure of 8 mmHg was utilized in all the VATS cases ***(Fig. 1*** and ***Fig. 2***.

The entire hemithorax was carefully examined with particular attention to the mediastinum and pleural cavities, followed by evaluation of macroscopic capsular invasion. No pleural symphsis was encountered in all selected cases. Any sign of local vessel or organ invasion (Masaoka III or more advanced stage) was an indication for conversion to an open procedure that was excluded in the present study. The borders of resection were the diaphragm caudally, the thyroid gland orally, and the phrenic nerves laterally. The Harmonic Scalpel (Ethicon Endo-Surgery, Cincinnati, OH, USA) was widely used in the VATS group. The technique was slightly modified for resection of a thymoma compared to that of non-thymoma. We preferred the right side approach when the mass was more or less in the midline or right-sided (no obviously left-sided thymoma was presented in the VATS group in the study). The non-tumorous part of the thymus and perithymic fat were always dissected first and used for grasping and traction while dissecting the tumor to minimize the risk of capsular damage. A two-layered retrieval bag was needed for piece-by-piece removal of all the specimen.

### Surgical technique of trans-sternal thymectomy (T_3b_)

For the T3b group, the patients were placed in the supine position under general anesthesia with a single-lumen endotracheal intubation. Thymic tissues with pericardial fat were removed radically by conventional instruments through median sternotomy (the extent of radical removal was similar to that of VATS).

### Statistical analysis

Categorical variables were presented by number of cases and analyzed by Pearson's chi-square test or Fisher's exact test. Continuous variables were expressed as mean±standard deviation (SD) and analyzed by independent two-sample *t* test. The impacts of prognostic factors (CSR and ER) were estimated by the Kaplan-Meier method. A log rank test was used to find the differences of the CSR and RR values between the two groups. *P* values of 0.05 or less were considered statistically significant.

## RESULTS

In the study, 15 patients underwent VATS thymectomy and 18 underwent trans-sternal thymectomy. The mean age at surgery was 51.1 y (range: 31-78 y, 18 males and 15 females), and the mean preoperative duration of MG (from the onset of MG to surgery treatment) was 7.2 months (range: 20 d to 48 months). There was no conversion to median sternotomy for VATS patients. The two groups did not significantly differ in demographic and preoperative data (***Table 1***), including gender, age at surgery, body mass index (BMI), duration of MG, severity of the MG symptoms, dosage of pyridostigmine intake and proportion of steroid use for patients. Intra- and postoperative data of the VATS and T3b group are also summarized in ***Table 1***. No statistically significant difference was obtained in the variables of duration of pleural drainage, volume of pleural drainage, length of intensive care unit (ICU) stay and postoperative hospital stay, the maximum diameter of thymoma measured by pathologists, and distributions of Masaoka stage of thymoma. Meanwhile, the VATS group had favorable outcomes compared with the T3b group in the variable of intraoperative blood loss (*P* < 0.001), but more time was needed for VATS thymectomy (*P* = 0.001). The proportions of type A, AB, B1, B2, and B3 thymoma, and thymic carcinoma were 20.0%, 26.7%, 20.0%, 26.7%, 6.6%, and 0% in VATS and 0%, 27.8%, 22.2%, 33.3%, 5.6%, and 11.1% in the T3b group, respectively.

**Table 1:** Demographic and clinical data of the VATS and T3b group

Variables	VATS group (*n* = 15)	T3b group (*n* = 18)	*P* value
Sex (female: male)	8:7	7:11	00.632
Age (year)	54.20±11.89	48.56±8.97	0.130
BMI (kg/m^2^)	24.51±2.440	23.73±3.37	00.461
Duration of MG (month)	7.58±8.27	006.77±11.97	0.827
MGFA classification (%):	5(33.3)	4(22.2)	00.748
I	5(33.3)	4(22.2)	0.748
IIa	2(13.3)	1(5.6)0	00.868
IIb	1(6.7)0	2(11.1)	0.868
IIIa	1(6.7)0	0(0) 0.	00.926
IIIb	3(20.0)	6(33.3)	0.643
IVa	2(13.3)	3(16.7)	00.825
IVb	1(6.7)0	2(11.1)	0.868
V	0	0	NA
Pyridostigmine (mg/d)	198.00±80.020	195.00±77.86	0.914
Steroid use (%)	1(6.7)0	3(16.7)	00.733
Duration of surgery (minute)	202.33±53.110	141.78±30.74	0.001*
Volume of intraoperative blood loss (mL)	98.67±62.78	0225.00±101.82	< 0.001*
Duration of pleural drainage (day)	3.47±0.92	03.56±1.15	0.810
Volume of pleural drainage (mL)	394.00±151.98	0409.72±159.03	00.775
Duration of ICU stay (hour)	27.80±23.15	018.28±18.45	0.198
Duration of postoperative hospital stay (day)	10.60±5.110	12.22±3.64	0.29
Total morbidity (%)	4(26.7)	6(33.3)	0.972
Prolonged intubation**	1(6.7)0	2(11.1)	00.868
Pneumonia	2(13.3)	3(16.7)	0.825
Pleural effusion	2(13.3)	1(5.6)0	00.868
Blood transfusion	2(13.3)	5(27.8)	0.560
Arrhythmia	1(6.7)0	0(0)0.0	00.926
Lesion of phrenical nerve	0	0	NA
Bleeding	0	0	NA
Wound infection	0	0	NA
MG crises	1(6.7)0	1(5.6)0	00.549
Mortality	0	0	NA
Distribution of Masaoka stage (stage I:II)	6:9	10:8	00.589
Adjuvant therapy (%)	5(33.3)	9(50.0)	0.541
Recurrence of thymoma	0	0	NA

*Significant differences were acquired in the two variables (*P* < 0.05); **Prolonged intubation is defined as intubation time of more than 48 h after surgery. BMI: body mass index (kg/m^2^); ICU: intensive care unit; MG: myasthenia gravis; MGFA: MG foundation of America; VATS: video-assisted thoracoscopic surgery; NA: not available.

There was no significant difference in perioperative morbidity and mortality in both groups. No mortality occurred in the course of hospitalization. Postoperative morbidity was 26.7% and 33.3%, respectively, for the VATS and T3b group and respiratory complications (mainly including prolonged intubation, pneumonia and pleural effusion) were common, but they were no significant differences between the two groups. Two VATS (13.3%) and five T3b patients (27.8%) required blood transfusion (*P* = 0.560). Only one patient after VATS with suspected phrenic nerve injury was asymptomatic except for an elevated left-sided hemidiaphragm for three days, which was excluded from the present evaluation. There was one patient with postoperative myasthenic crisis in each group, and both needed intubation support and discharged with a longer hospitalization time.

**Fig. 3:**
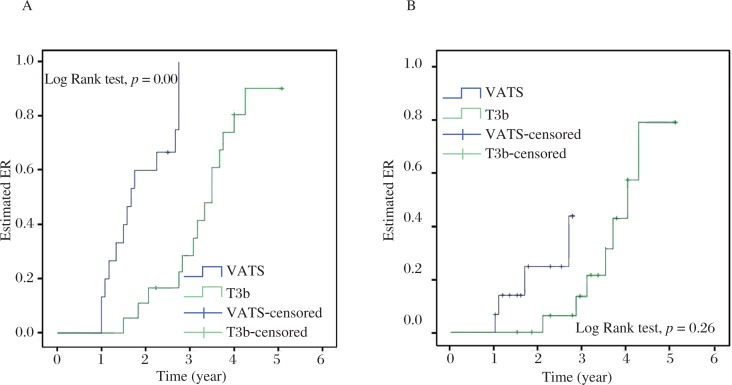
Estimated ER (A) and CSR (B) by Kaplan-Meier method in the VATS and T3b group. ER: effective rate; CSR: complete stable remission.

All 33 patients were followed up for 12 to 61 months in the study. The cumulative probabilities of reaching CSR and ER were 26.7% (4/15) and 93.3% (14/15) in the VATS group, which had a significantly higher CSR and ER than those in the T3b group (*P* = 0.026 and *P* = 0.000, respectively). Estimated CSR and ER calculated by the Kaplan-Meier method are illustrated in ***Fig. 3***. Only 1 patient was lost to follow-up and 1 patient died from myasthenic crisis 1 year postoperatively in the T3b group. No thymoma recurrence or even port site recurrence was detected and no exacerbation occurred in any of the patients after CSR or ER was achieved.

## DISCUSSION

VATS was introduced in 1992 as a minimally invasive option for the treatment of MG. During the following years, VATS thymectomy gained widespread acceptance in the treatment of MG, especially for non-thymoma-associated MG. However, few published literature were available to exclusively report the effect of VATS thymectomy on the treatment of T-MG. It is generally accepted that "complete" thymectomy should be the goal of treatment of MG, that is, complete en-bloc resection of the tumor and removal of the upper cervical poles and all surrounding mediastinal fat with more attention to the ectopic thymus in the aortopulmonary window region, around the phrenic nerves, the superior vena cava, and the extension deep behind the left innominate vein. Due to lack of proper, randomized, prospective clinical investigation, whether a VATS thymectomy has the same operative quality as median sternotomy is still controversial. Unlike VATS thymectomy performed as the preferred procedure for non-thymoma-associated MG[7]–[9], there are, at least, three major uncertainties in the treatment of T-MG.

The first major concern is the preferred approach of the VATS procedure. Following the approach first proposed by Yim[10], a right-sided VATS approach was adopted that continues to be used now. As more experience has been gained with the right-sided approach, it has become the preferred approach. The advantages of the right-sided approach include greater maneuverability of the scope and instruments in the relatively greater expanse of the right pleura cavity and easier identification of the innominate vein because of easy visualization of the superior vena cava as a landmark for identification of this structure. An additional reason is the advantage for right-handed surgeons to access the cervical area from the right side. On the other hand, Mineo et al. [11] reported the surgical details of the left-sided approach as well as the advantage of performing adjuvant pneumomediastinum to facilitate the dissection of both thymus and the perithymic fatty tissue. Although we agree that the thymus can be safely approached by VATS from both sides, we prefer access from the right side, except in cases of obvious left-sided thymoma presented in preoperative CT scan.

The second concern is the feasibility of gaining a good visualization for an endoscopic operation. In order to gain a good visualization, some authors proposed "sternum lifting" technique[12]. They extended this method to patients with thymoma of stage I, II and some stage III (invasion to the lung and pericardium) and obtained an unquestionably functional and cosmetic advantage for patients. Others emphasized the utilization of adjuvant pneumomediastinum with carbon dioxide insufflation, which was used for "opening up" the mediastinal and cervical area, to facilitate the dissection of both thymus and the perithymic fatty tissue[13]. Since 2005, we have utilized adjuvant pneumomediastinaum and performed complete thymectmy using a right-sided approach for VATS in MG patients in our department, and no evidence of adverse effect of thoracic carbon dioxide insufflation on the patients' hemodynamics was present intraoperatively[14]. As expected in our practice, we found that this technique offers several advantages. Firstly, it may be less disfiguring and no scars are left compared to other techniques, such as the sternum lifting method, while still providing satisfactory visualization of the thoracic cavity. Secondly, the left sides of the superior pole of the thymus and pericardiophrenic angle of the perithymic fatty tissue can be clearly visualized and thoroughly mobilized (***Fig. 1*** and ***Fig. 2***). Thirdly, also most importantly, the left phrenic nerve can be better visualized and protected during the course of clearance of fatty tissue around the phrenic nerve, because the left lung moves leftward secondary to the pneumomediastinum, which ensures adequate room for surgical maneuvers subsequently. In addition, by making good use of these incisions (e.g., by replacing thoracoscopy through the anterior incision), all the ectopic thymus was not difficult to remove completely unless the thymoma is too large for the surgeon to visualize the contralateral side, which tends to indicate bilateral VATS or trans-sternal approach.

The third is oncological considerations for manipulations of thymoma, which are essential to minimize the risk of breaking the tumor capsule and tumor seeding. The role of VATS as a therapeutic modality has rapidly expanded, and several authors have reported the use of VATS for the treatment of anterior mediastinal tumors, including thymectomy for MG. Cheng[15] emphasized that resection of stage II thymoma by VATS met both the anatomic and surgical requirements of successful treatment with the careful manipulation of tumor tissue and protective measures (protection bags and introduction trocars) may be responsible for pleural and parietal implantations by seeding cells. Agasthian and his colleagues[16] showed a similar technique of thymoma resection described as "no-touch, tumor-last" to ours. If the tumor is dissected first, it makes dissecting the rest of gland not only difficult but also oncologically hazardous.

Up to now, there is no consensus on the appropriate size required to permit minimal invasive thymectomy. There are mainly two minimal invasive approaches to thymectomy so far: the first is VATS and its variant including sternum-lifting, use of subxiphoid incision and robotic-assisted surgery, and the second is the transcervical approach with or without video assistance[17]. From the viewpoint of resectability, invasion to the surrounding tissues, especially to the left innominate vein, is more important than the size of the tumor[18]. Most authors restrict the indication of minimal invasive thymectomy to the early thymoma smaller than 5 cm in size and with no invasiveness[10],[11],[16],[19],[20]. Others offered the VATS procedure using the Harmonic Scalpel to patients with MG and encapsulated thymomas of 6 cm in size[21].

The VATS approach provides an obvious advantage of the trans-sternal technique in the aspect of volume of intraoperative blood loss, but at the cost of prolonged operative time. The latter could be interpreted by the more extent of difficulty of manipulation of thymomas with VATS[20]. There was no statistically significant difference in the variables of duration of pleural drainage, volume of pleural drainage, duration of ICU stay and postoperative hospital stay in both groups, which may be attributed to the small samples evaluated in this study.

As previous studies comparing different minimally invasive operative techniques in the management of MG with thymomas have shown that CSR ranges from 11.3% to 37.0% and ER from 60.2% to 100%, depending on different follow-up periods, preoperative severity of MG, and proportion of T-MG in the study cohort (***Table 2***)[10],[11],[16],[19],[20],[22]-[24]. In our study, the cumulative probability of reaching CSR was 26.7% and ER was 93.3% for the VATS group in our follow-up periods. Patients in the VATS group illustrated better CSR and ER results than the trans-sternal approach, which were probably due to more severe MG patients in the T3b group than those in the VATS group prior to surgery. Meanwhile, there has been no literature available reporting the advantage of VATS technique in neurological outcomes comparing with conventional trans-sternal approach so far.

**Table 2:** Results of VATS thymectomy for MG with thymoma: comparisons with published outcomes

Author	Number of myasthenic patients*	Presence of thymoma [n(%)]**	Follow-up time	CSR (%)***	ER (%)***	Selection criteria of thmoma for VATS thymectomy
Yim et al (1995)[10]	8	3(37.5)0	Mean: 10 months	NA	100	No invasion, stage I thymomas, 3.5–4 cm in diameter
			Range: 2-20 months			
Mack et al (1996)[19]	33	6(18.2)0	Mean: 23.4±11.7 months	18.0	87.9	Stage I thymoma
			Range: 4–47 months			
Mineo et al (2000)[11]	31	4(12.9)0	Mean: 39.6±15 months	36.0	96.0	1.5–3 cm in maximal size, nosign of invasiveness
			Range: 16–75 months			
Hsu et al (2004)[20]	27	6(22.2)0	Mean:18.5months	37.0	NA	4 cases of stage I, 2 cases of stage II.
			Range: 6–30 months			Recommend:large thymoma (>2 cm in diameter) can be resected by thoracoscopic approach
Maggi et al (2008)[23]	71	71(100)0	Mean: 7.69±6.0 years	11.3	NA	NA
			Range: 1.1–32.2 years			
Meyer et al (2009)[23]	48	4(8.3)00	Mean: 6.0±4.0 years	34.9	60.2	NA
Agasthian et al (2010)[16]	61	32(52.5)	Mean: 4.9 years	21.0	74.0	Well-encapulated thymoma (range: 10–90 mm in size), 7 cases of stage III(mean size: 25 mm) also included
			Range: 1.9–10 years			
Yu et al (2012)[24]	219	67(30.6)	Range: 4 months-9 years	28.3	71.6	NA
Current study	15	15(100)0	Range: 12–33 months	26.7	93.3	Stage I and II, tumor diameter: 4.03±2.22 cm, Range: 0.5–9.5 cm

*Number of myasthenic patients for VATS thymecromy in the study cohort; **Number of myasthenic patients with thymoma for VATS thymectomy in the study cohort; ***Estimated CSR and ER of myasthenic patients for VATS thymectomy. NA: not available; CSR: complete stable remission; ER: effective rate; MG: myasthenia gravis; VATS: video-assisted thoracoscopic surgery.

The patients in our study were not grouped randomly but according to their preferences. However, to minimize any variability of the two groups, the patients of the two groups were comparable in age, sex ratio, duration of MG prior to surgery, severity of symptoms, and even the diameter of thymoma. Despite a small series and short-term follow-up, we preliminarily conclude that VATS thymectomy for the treatment of MG with stage I and II thymoma is technically feasible but deserves further investigation, as late recurrences are described up to 15 years after initial resection[25]. We presently believe that VATS thymectomy should be performed only under strict protocol setting by surgeon experienced with VATS. Further investigation with long-term follow-up would clarify the role of VATS thymectomy in thoracic surgery.
